# Sclerosing Angiomatoid Nodular Transformation: Laparoscopic Splenectomy as Therapeutic and Diagnostic Approach at the Same Time

**DOI:** 10.1155/2018/7020538

**Published:** 2018-05-08

**Authors:** Calogero Cipolla, Ada Maria Florena, Gabriella Ferrara, Riccardo Di Gregorio, Elettra Unti, Antonino G. Giannone, Luigi A. Lazzaro, Giuseppa Graceffa, Gianni Pantuso

**Affiliations:** ^1^Department of Surgical, Oncological and Oral Sciences, Division of General and Oncological Surgery, University of Palermo, Palermo, Italy; ^2^Department of Sciences for Promotion of Health and Mother and Child Care, Anatomic Pathology, University of Palermo, Palermo, Italy

## Abstract

**Introduction:**

Sclerosing angiomatoid nodular transformation (SANT) of the spleen is a rare benign vascular lesion with unknown etiopathogenesis and with definite features of imaging, histopathology, and immunohistochemistry. It was first described by Martel et al. in 2004, and to date, only 151 cases have been reported.

**Case Description:**

We report a case of SANT of the spleen detected in a 66-year-old Caucasian, without comorbidities, presented to our department with epigastric pain. We, also, presented a review of the literature.

**Conclusions:**

SANT is a benign incidentally vascular condition in the majority of cases. The wide age and gender distribution in our review is in accordance with that in previous studies in English literature. In our opinion, splenectomy is the choice treatment because it is at the same time diagnostic and therapeutic in a definitive way.

## 1. Introduction

Sclerosing angiomatoid nodular transformation (SANT) of the spleen is a rare benign vascular lesion with unknown etiopathogenesis and with definite features of imaging, histopathology, and immunohistochemistry. It was first described by Martel et al. in 2004 [1], and to date, only 151 cases have been reported.

However, real incidence and prevalence are still unknown. In this paper, we report a case of SANT of the spleen managed in our institute and we present a review of the literature.

## 2. Case Report

**Figure 1:**
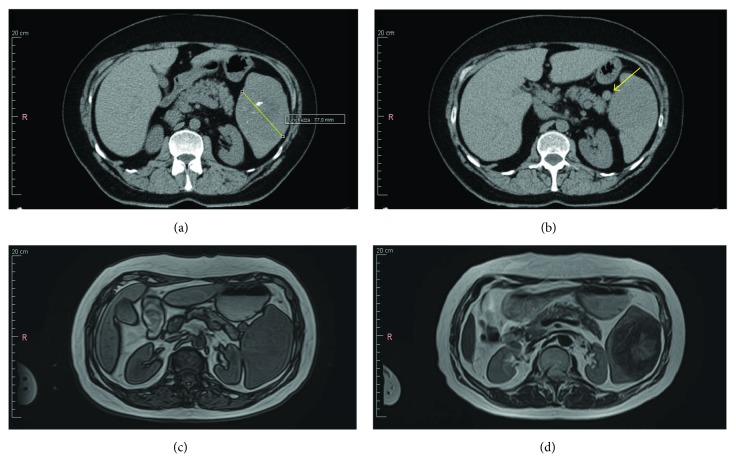
SANT on the imaging. (a) CT: hypodense lesion with a 77 mm maximum diameter and calcifications in the context. (b) CT: centimetric accessory spleen. (c) MRI: lesion (6.3 cm × 8.2 cm) characterized by heterogeneous intensity, iso-hypointense in T1. (d) MRI: a signal hypointense of the lesion in T2 with a centrally scattered hyperintense signal on the fat saturated precontrast phase.

A 66-year-old Caucasian woman, without comorbidities, presented to our department with epigastric pain. Abdominal examination did not evidence any other clinical signs. Hematological parameters reported an increase in PCR values, with no other pathological findings. The abdominal ultrasonography demonstrated a regular hypoechoic nodular formation at the lower pole of the spleen with an 8 cm maximum diameter, with calcification spot. Computed tomography scan confirmed the hypodense lesion presence, with a 77 mm maximum diameter, and calcifications in the context (Figure 1(a)). In addition, a centimetric accessory spleen adjacent to the lower pole was detected (Figure 1(b)). MRI study showed a massive lesion (6.3 cm × 8.2 cm) characterized by heterogeneous intensity, iso-hypointense in T1 (Figure 1(c)), predominantly hypointense in T2 with a centrally scattered hyperintense signal on the fat saturated precontrast phase (Figure1(d)). The early arterial phase evidenced peripheral enhancement with central radial progression, on the late phase images.

**Figure 2:**
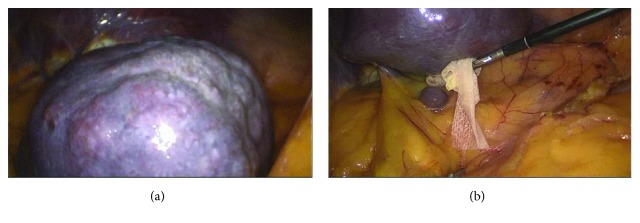
Laparoscopic view of the spleen. (a) The mass at the lower pole of the spleen. (b) Accessory spleen.

We decided to proceed with surgical therapy, without making a spleen biopsy, and we opted for a laparoscopic splenectomy, because of diagnostic uncertainty due to unspecific features of imaging not excluding the malignant lesion nature. During abdominal cavity exploration, we identified the mass at the lower pole of the spleen (Figure 2). The spleen was completely placed in a specific bag, and, after centimetric accessory spleen removal, we sent it to the Department of Pathology of our hospital structure for histological examination. The postsurgery period had normal results except for a single episode of atrial fibrillation. We also administered a pneumococcal vaccine to prevent any postsplenectomy infections and discharged the patient 8 days after the surgery without any complications.

## 3. Pathologic Findings

**Figure 3:**
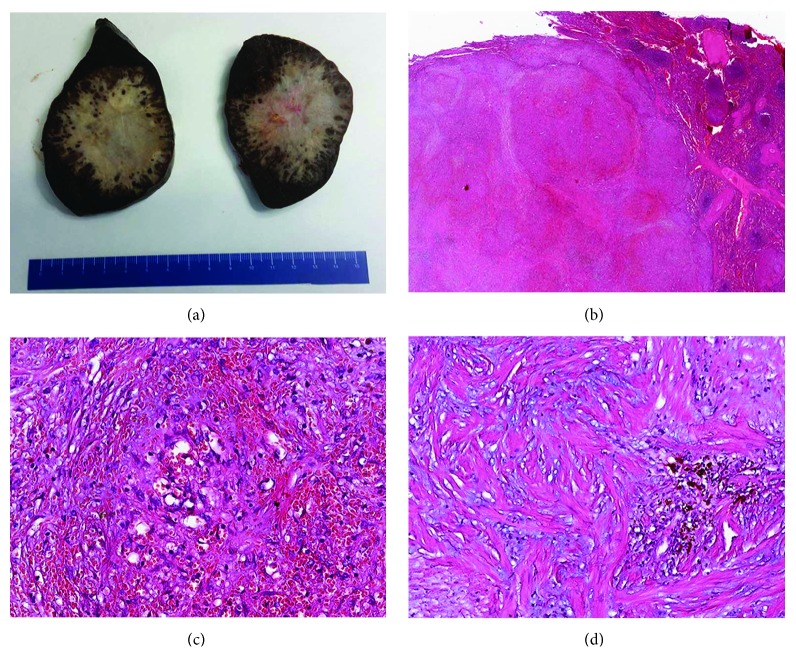
Histopathological findings. (a) On gross examination, the cut surface shows a solitary, well-circumscribed but unencapsulated lesion; at the periphery, multiple dark-brown nodules are interspersed with the fibrotic stroma. (b) At low magnification, the lesion consisted of multiple angiomatoid nodules in a fibrotic stroma, with sharp demarcation from the adjacent splenic parenchyma (right). (c) Vascular component within nodules composed by small-sized blood vessels, with slit-like, round, or irregular shape; an incomplete fibrinoid rim was present at the periphery of this angiomatoid nodule. (d) The internodular tissue consisted of dense fibrous tissue with myxoid areas with siderophages and scattered plump myofibroblasts, plasma cells, and lymphocytes. Original magnifications—b: ×10; c and d: ×200.

**Figure 4:**
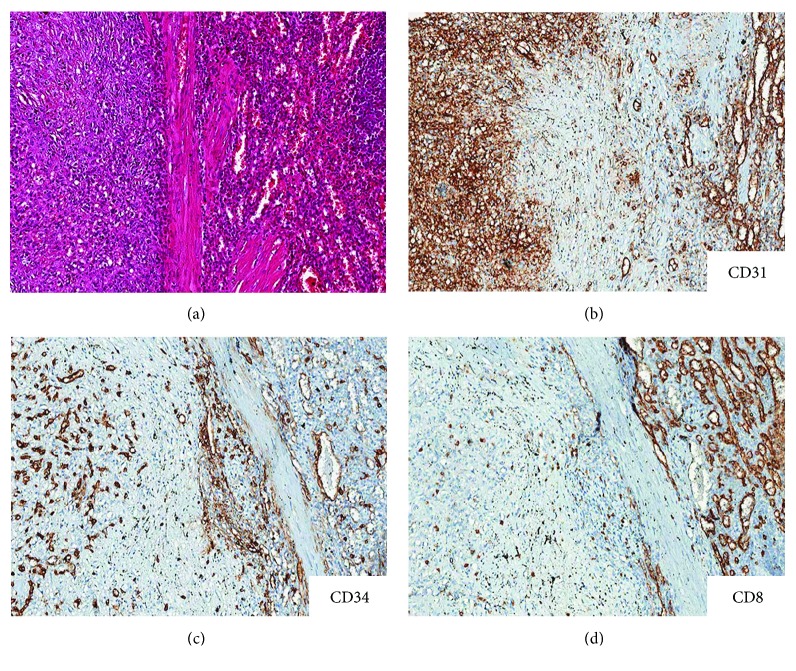
Comparison of the lesion (a, c) with the adjacent spleen parenchyma (b, d). The overall phenotype of lesional vessels differed from that of spleen parenchyma. The blood vessels within the lesion showed three main immunophenotypes: CD34+/CD8–/CD31+ capillaries, CD34–/CD8–/CD31+ small veins, and rarer CD34–/CD8+/CD31+ sinusoids. Original magnifications—a–d: ×100.

The spleen measured 12.5 × 10 × 7 cm and weighted 480 g; the lower pole was occupied by an unencapsulated nodular lesion, 7 × 7.2 × 6 cm, with multilobular architecture and well-defined and stellate edges with infiltrative margins. The lesion was firm, with grayish to white-tan color (Figure 3(a)). On low magnification, histologic sections showed vascular nodules of variable sizes, sometimes coalescent, surrounded by a rim of concentric fibrous tissue (Figure 3(b)). The vascular component within nodules was composed by variably sized blood vessels, with slit-like, round, or irregular shape (Figure 3(c)). The vascular spaces were lined by plump endothelial cells with occasional spindle or ovoid cells. Nuclear atypia was absent; mitotic figures were inconspicuous (<1/50 HPF). The internodular tissue consisted of dense fibrous tissue with myxoid areas and scattered plump myofibroblasts, plasma cells, lymphocytes, and siderophages (Figure 3(d)). The surrounding splenic parenchyma showed prominent vascular congestion and blood extravasation. Blood vessels within the lesion showed three main immunophenotypes: CD34+/CD8–/CD31+ capillaries, CD34–/CD8+/CD31+ sinusoids, and CD34–/CD8–/CD31+ small veins (Figure 4). The proliferation index, assessed with Ki67, was lower than 2%. Plasma cells were positive for CD138 with polytypic expression of m and l light chains but were negative for IgG4. CD68 highlighted siderophages and sinusoidal macrophages. The immunohistochemical stains for HHV8, EBV-LMP1, and ALK1 were negative. All these features were consistent with sclerosing angiomatoid nodular transformation of the spleen.

## 4. Discussion

SANT of the spleen first described by Martel et al. [1] in 2004. They exposed a series of 25 patients who presented the same clinical, imaging, histopathology, and immunohistochemistry features. In this paper, we reported a case of SANT cured in our surgical unit and reviewed retrospectively clinical cases of 151 patients in English literature, starting with the 128 cases reported by Cao et al. [1–3] in 2015 and expanding this research with 23 cases reported in other studies from October 2011 to the present (Table 1).

In this study, we reported a total of 152 cases of SANT (including this case) that included 73 males (48.02%) and 79 females (51.97%). The age range is 3 to 82 years old, with a mean age of 45 (45.44) years which is a little bit inferior to the data reported by Cao et al. [3]. The ratio of male to female was 1 : 1.08, with an incidence generally comparable between both genders according to the previous analysis.

Our case could be added to the 144 cases of solitary SANT already described in literature, whereas only 7 cases were multifocal SANT [3–5]. In our review of the cases in English literature, 109 of these 151 cases (72.18%) did not present any clinical symptoms and accidentally discovered the lesion during imaging controls. In the remaining patients, the main symptom was abdominal pain (*n* = 39). Other symptoms were a palpable abdominal mass, cytopenias, flank pain, pelvic pain, fever, nosebleed and anemia [6, 7], vomiting, pruritus in the lower limbs [8], and weight loss [9]. There were two reported deaths in the Martel's cases [1]: a 56-year-old woman who died of disseminated lung adenocarcinoma and a 46-year-old man with concurrent bronchogenic squamous cell carcinoma who died of sepsis post splenectomy. In our case, abdominal MRI excluded a concomitant neoplasm and pneumococcal vaccination was used to reduce the risk of infection. Currently, pathognomonic detection for the imaging diagnosis of SANT does not exist [10]. The general features are peripheral enhancing radiating lines and rim enhancement of the lesions evaluated with contrast-enhanced CT or MRI during the arterial or portal venous phases. Gutzeit et al. [11] referred to this pattern of enhancement as a “spoke wheel” appearance on contrast-enhanced ultrasound, CT, and MRI. Furthermore, Gutzeit proposed the use of contrast-enhanced ultrasonography to diagnose SANT, but there are still a few studies about it. SANT must be histologically distinguished from inflammatory pseudotumor (IP), hamartomas, and the vascular proliferations of the spleen, such as littoral cell angioma, capillary and venous hemangioma, hemangioendothelioma, lymphangioma, angiosarcoma, and nodular transformation of the splenic red pulp [12] (desmoplastic response to metastatic carcinoma). IP represents one of the main differential diagnosis; the two lesions can often share some architectural similarities, especially in the stromal component, but the lack of multinodular architecture, together with the negativity for ALK1, can aid to rule out the diagnosis of IP [4]. Splenic hamartoma [13] is a tumor-like lesion composed of red pulp elements with disordered architecture, without the typical angiomatoid multinodular structure, observed in SANT. Moreover, the phenotype of blood vessels in splenic hamartoma differs from SANT. The vascular spaces of SANT comprise various nature vessels (capillary, sinusoids, and veins) with a specific phenotype; on the opposite, vascular tumors show vessels with a monotonous phenotype, with variable features of malignancy, ranging from hemangioma [14] and lymphangioma to hemangioendothelioma and angiosarcoma. Although rare, SANT is a diagnostic entity to be taken into account among splenic nodular lesions, with well-established diagnostic features. According to recent studies, percutaneous image-guided CNB of the spleen is becoming safe and effective in tissue diagnosis results [6]. There is a debate about whether an asymptomatic patient should be subjected to a surgical procedure to obtain diagnosis. Most of the published cases get to the diagnosis after splenectomy. Despite that Weinreb et al. [15] suggest that good core biopsy can be used to distinguish SANT from other lesions, splenic biopsy has been employed rarely (only 2 to 23 cases in Table 1). Anyway, there is a significant intraperitoneal [6, 15] dissemination risk if the biopsied lesion proves to be angiosarcoma and have other complications (splenic rupture and bleeding). The etiopathogenesis is still unknown, but several causes are hypothesized: Epstein-Barr virus association [16], red pulp abnormal transformation due to stromal proliferation, or hamartomas/inflammatory pseudoneoplasm final stage [6, 17]. Finally, in recent studies [18], it is noted that SANT proliferation could be associated to the typical sclerosing injuries of the disorder related to immunoglobulin G4 (IgG4).

SANT is a benign incidentally vascular condition in the majority of cases. The wide age and gender distribution in our review is in accordance with the previous studies in English literature. In our opinion, splenectomy is the choice treatment because it is at the same time diagnostic and therapeutic in a definitive way.

## Figures and Tables

**Table 1:** Clinical features of 23 cases of SANT reported in literature.

Author	*N*	Age	Gender	Clinical features	Spleen weight	Gross features	Follow-up	Referring dx	Concurrent disease	Core needle biopsy
Lee et al. [19]	1	38	F	Upper abdominal discomfort associated with nausea	—	5.1 × 4.7 cm, multiple tan well-defined nodules ranging from 0.8 cm to 1.2 cm	—	SANT	—	—
Murthy et al. [6]	1	56	M	Left upper quadrant pain and mild anemia	578 g	17.5 × 11 × 9.5 cm, central bulky stellate mass of white compact tissue (5 × 4 × 4.5 cm)	NED, 2 years	SANT	—	Yes
Kakisaka et al. [7]	1	36	M	Nosebleed and anemia	692 g	13 cm in diameter	—	SANT	—	—
Yoshimura et al. [20]	2	56 57	M M	Follow-up unenhanced CT Medical checkup	—	Largest dimension of 6.5 cm 5.6 cm	—	SANT SANT	—	—
Zhang et al. [14]	1	3	M	Abdominal pain and vomiting 2 hours after being involved in a car accident	—	Solitary lesion, measuring 4 × 5 × 3 cm	NED, 20 months	SANT	—	—
Corrado et al. [8]	1	37	F	Pruritus in the lower limbs	—	13 × 7 × 6 cm, with a solitary, well-circumscribed mass, measuring 5 cm in greatest diameter, with a multinodular fibrotic cut surface	NED, 10 months	SANT	Homozygous mutation of methylene tetrahydrofolate reductase (MTHFR) and polymorphism of A1298C presented in the 19th week of gestation	—
Lim et al. [21]	1	39	M	Incidental findings	277.2 g	11.0 × 9.0 × 7.0 cm; a well-circumscribed tumor measuring 5.0 × 4.5 × 4.5 cm was identified	—	SANT	—	—
Bagul and Sen [22]	1	24	M	Pain in the left hypochondrium	290 g	12 × 10 × 8 cm, well-circumscribed tense lesion, dark red in color with interspersed stellate white projections	—	SANT	—	—
Metin et al. [9]	1	21	M	Fatigue, weight loss, and abdominal pain for 4 months	200 g	11 × 8 × 7 cm, well-demarcated nodular lesion 3 × 2.5 × 2.3 cm in size at the hilum	—	SANT	—	Yes
Zhou et al. [23]	4	40 50 65 59	F M M F	Incidental imaging studies	— — — —	9.5 × 6.5 × 5.5 cm 8 × 6.5 × 5 cm 6 × 4 × 3 cm 5.5 × 5 × 4.5 cm	6 months, NED 9 months, NED 48 month, NED 8 months, NED	SANT	Left papillary thyroid carcinoma with metastasis to lymph nodes; multiple hepatic cysts; splenic mass Hypertension; hepatitis A; multiple hepatic cysts; splenic mass Hypertension; multiple hepatic cysts; nephric and splenic mass Hypertension; diabetes; left nephric cysts; gallbladder polyps; mild fatty liver; splenic mass	—
Giorlandino et al. [24]	1	71	M	Incidental imaging studies	120 g	10 × 7 × 2.5 cm, a 7 cm mass with an exophytic growth and a broad-based implant was observed at the superior pole	−1 year, NED	SANT	—	—
Singh et al. [25]	1	36	M	Routine screening for abdominal pain	100 g	Nodule measuring 1.5 cm in maximum dimension, located at the superior pole	—	SANT	—	—
Kim et al. [26]	1	23	F	Incidental imaging studies	—	Solitary mass, measuring 5.2 × 4.5 cm	—	SANT	—	—
Nagai et al. [18]	1	33	M	Incidental finding during follow-up after adrenalectomy for primary aldosteronism	—	Well-demarcated, solitary lesion measuring 7 × 6.5 cm	—	SANT	—	—
Wang et al. [27]	1	29	M	1-year history of left upper quadrant and back discomfort	—	Well-circumscribed mass measuring 7 cm in diameter	13 months	SANT	—	—
Cafferata et al. [17]	1	57	M	Mild left upper abdomen discomfort	448 g	A solitary, unencapsulated lesion (main diameter: 7 cm) with round, well-circumscribed borders	10 months	SANT	Focal nodular hyperplasia and hemangioma of the liver	—
Aracil León et al. [28]	1	55	M	Incidental finding during follow-up after renal abscess is resolved	139 g	A solitary, unencapsulated lesion (5.5 × 4 cm)	15 months	SANT	—	—
Martínez Martínez et al. [29]	1	57	F	Epigastric abdominal pain and left hypochondrium pain	—	Well-delimited lesion in the addendum splenic tissue, 9 cm long from the major axis	—	SANT	Depression	—
Imamura et al. [30]	1	37	F	Incidental imaging studies	280 g	Well-circumscribed 4.5 × 4 cm mass	12 months	SANT	—	—
